# Prevalence of enamel defects in primary and permanent teeth in a group of schoolchildren from Granada (Spain)

**DOI:** 10.4317/medoral.18580

**Published:** 2012-12-10

**Authors:** Maria J. Robles, Matilde Ruiz, Manuel Bravo-Perez, Encarnación González, Maria A. Peñalver

**Affiliations:** 1 DDS, Postgraduate Student. University of Granada, Campus de Cartuja, Colegio Máximo s/n, Granada, Spain; 2 DDS, PhD: Assistant Professor, Department of Paediatric Dentistry, School of Dentistry. University of Granada, Campus de Cartuja, Colegio Máximo s/n, Granada, Spain; 3 DDS, MD, PhD: Professor, Department of Preventive Dentistry, School of Dentistry.University of Granada, Campus de Cartuja, Colegio Máximo s/n, Granada, Spain; 4 DDS, PhD: Associate professor, Department of Paediatric Dentistry, School of Dentistry. University of Granada, Campus de Cartuja, Colegio Máximo s/n, Granada, Spain; 5 DDS, MD, PhD: Associate professor, Department of Paediatric Dentistry, School of Dentistry. University of Granada, Campus de Cartuja, Colegio Máximo s/n, Granada, Spain

## Abstract

Objective: The purpose of this study was to determine and compare the prevalence and presentations of developmental defects of the enamel (DDE) in the primary and permanent dentitions of a group of healthy schoolchildren residing in Granada (Spain). Study Design: A total of 1,414 healthy schoolchildren were examined using modified DDE criteria for recording enamel defects. Results: The prevalence of DDE of any type was 40.2% in primary dentition and 52% in permanent dentition (p<0.033). Of the 31,820 primary and permanent teeth examined in the study, 699 (4.1%) primary and 1,232 (8.3%) permanent teeth had some form of DDE. Diffuse opacity was the most common type of DDE observed in primary teeth, and demarcated opacity in the permanent teeth. Enamel hypoplasia was the least prevalent defect in both dentition types. Conclusions: The study population showed a high prevalence of DDE in primary as well as in permanent dentition, reflecting the current increasing trend of this condition, which should be considered as a significant public health problem.

** Key words:**Developmental enamel defects, enamel hypoplasia, demarcated opacity, diffuse opacity.

## Introduction

Developmental defects of enamel (DDE) can be defined as any alteration resulting from diverse disturbances during the process of odontogenesis. They may be quantitative in nature, manifest as a deficient thickness of enamel or enamel hypoplasia (EH); or else qualitative (hypomineralization), presenting clinically as enamel opacity (EO)-in turn, either demarcated (DEO) or diffuse (DIO) (1).

These DDE can have a significant impact on oral health and esthetics, tooth sensitivity, and altered occlusal functions (2,3). Clinical treatment of children is a challenge for the dentist because sensitivity and pain may reduce the child’s cooperation. Moreover, these teeth may be difficult to anesthetize and entail a higher probability of repetitive failure of restoration (2-4). Enamel defects are now acknowledged as risk indicators for dental caries and erosion in children (5-6).

Most epidemiological studies show that the frequency of appearance of these defects is on the rise in practically all populations, underlining their clinical significance and relevance for public health initiatives (2,4,7-9). The occurrence of DEO associated with EH —a condition commonly called Molar Incisor Hypomineralization, on the increase as well (4,10). Studies report DDE prevalence in developed countries and healthy children to be in the range of 24% to 49% in primary dentition, and 9% to 63% in permanent teeth (2,8,9,11-21). In Spain, the only studies of DDE prevalence in permanent teeth are limited to recording lesions in incisors and/or first molars (4,22). No epidemiological study to date has focused on the prevalence of DDE in primary dentition among healthy Spanish schoolchildren; yet data are available for preterm and other medically compromised children (23,24).

The etiology of DDE is not completely clear. Genetic factors such as amelogenesis imperfecta are involved, along with environmental factors such as fluoride intake and medications, nutritional deficiencies, prenatal infections or chicken pox or other early childhood diseases (2,12,22,25). The importance of socioeconomic factors is evident, as DDE is much less prevalent in developed countries with good nutrition (12). Comparing clinical presentations of defects can provide insight into the response of ameloblasts to environmental insults in primary and permanent dentitions, and thereby facilitate the identification of etiological agents (2).

Given this background, the aim of our study was to examine a group of healthy Spanish children residing in a non-fluoridated community in order to assess and contrast the prevalence and presentations of developmental defects of the enamel in primary and permanent dentitions.

## Material and Methods

The sample population consisted of children of both genders, 3 to 12 years old, enrolled in four schools in the province of Granada (Spain). The schools were selected by simple random sampling, and classified as public, private and semi-private according to the administrative regime. The province of Granada does not have fluoridated drinking water (0.07 ppm of fluoride).

This study was approved by the ethics committee of the Department of Dentistry, University of Granada. After obtaining authorization from the schools´ principals, 1,717 letters of informed consent were distributed among parents to authorize a dental examination of the children, and to collect information about maternal health during pregnancy, the child’s overall pre-and postnatal health, and parental occupation in order to establish a relationship with socioeconomic status. Those schoolchildren with some physical or mental handicap, a history of serious illness or a chronic medical condition such as cardiac disease, or who had lived in a fluoridated community in the past were excluded from the study. Furthermore, teeth with more than two-thirds of the surface restored (including stainless steel crowns), badly decayed or fractured, were excluded, as were teeth with braces.

The measurement of socioeconomic status was based on the occupation of the head of the family, on an ordinal scale of I-V (high to low) (26).

The clinical examinations were conducted by a single trained dentist. The Modified DDE Index (FDI, 1992) (1) was used to diagnose and classify changes in the enamel of the teeth studied. Prior to commencement of the study, the examiner was trained in use of the DDE through color photographs showing typical enamel defects. Both intra- (one week between the two examinations) and inter-examiner (with an experienced expert) reliability were tested by repeating the dental examinations in 31 children, giving kappa values above 0.78, which is considered adequate according to the Landis and Koch scale (27).

**Figure 1:**
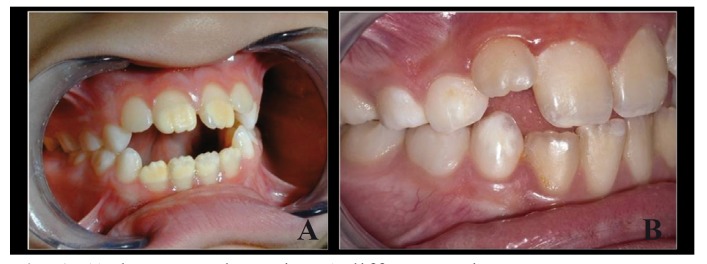
A) demarcated opacity B) diffuse opacity.

Before clinical examination, each child removed plaque with proper brushing, supervised by the explorer. The teeth were examined using artificial light, the tooth surface having been dried with a sterile gauze. Visible surfaces of all teeth were examined and scored for enamel defects according to the FDI criteria (1), which distinguish between defects that appear as changes in the translucency of enamel (EO), or as deficiencies in the quantity of enamel (EH). EO can be further categorized as DEO (Fig. 1A), if the borders of the lesion are well defined and DIO (Fig. 1B), if the lesion has no distinct borders. Enamel defects were differentiated from carious lesions by their clinical appearance and locations (usually not related to gingival margins or occlusal fissures) (2).

Descriptive statistical analysis (means, standard deviations, percentages) were calculated by means of SPSS 15.0 (SPSS Inc, Chicago, IL, USA). Associations (p-values) were calculated using SPSS 15.0 when the unit of analysis was the child, and using SUDAAN 7.0 (RTI, RTP, NC) when the unit was the tooth, to account for clustering (multiple teeth within the child). Statistical tests are clearly noted in Table and Figure footnotes.

## Results

There were 58 refusals to join in the study, whereas 1,659 children returned signed, informed consent from parents/guardians regarding participation. However, 39 of the latter did not attend school the day of the examination, and another 206 did not fulfill inclusion criteria, leaving a total of 1,414 (82.3%) participating children (705 males and 709 females).

 Table 1 gives the distribution of schoolchildren with and without DDE, according to type of dentition. Comparison of the preva-lence of DDE among groups was statistically significant (p<.036), being higher in the case of permanent dentition.

**Table 1:**
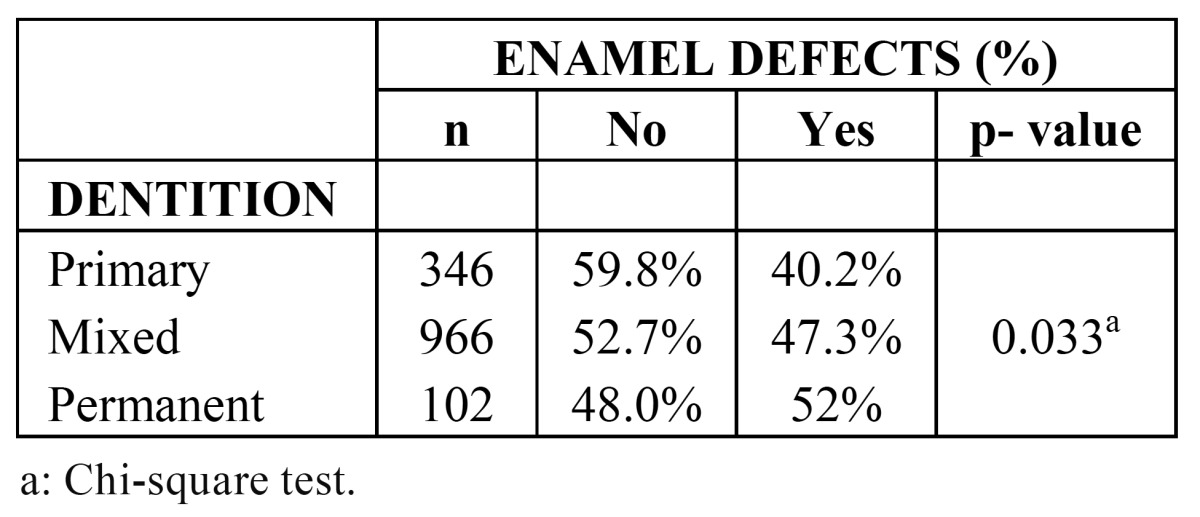
Number and distribution of schoolchildren with and without enamel defects (dde) according to type of dentition (n=1,414).

The data in  Table 2 show the frequency distribution of enamel defects according to age, gender, socio-economic level and school type. A greater prevalence of DDE was seen for male gender, age 8, and medium-low to low socioeconomic level (groups IV-V), in public schools, with statistically significant differences obtained in all the groups ( Table 2).

**Table 2:**
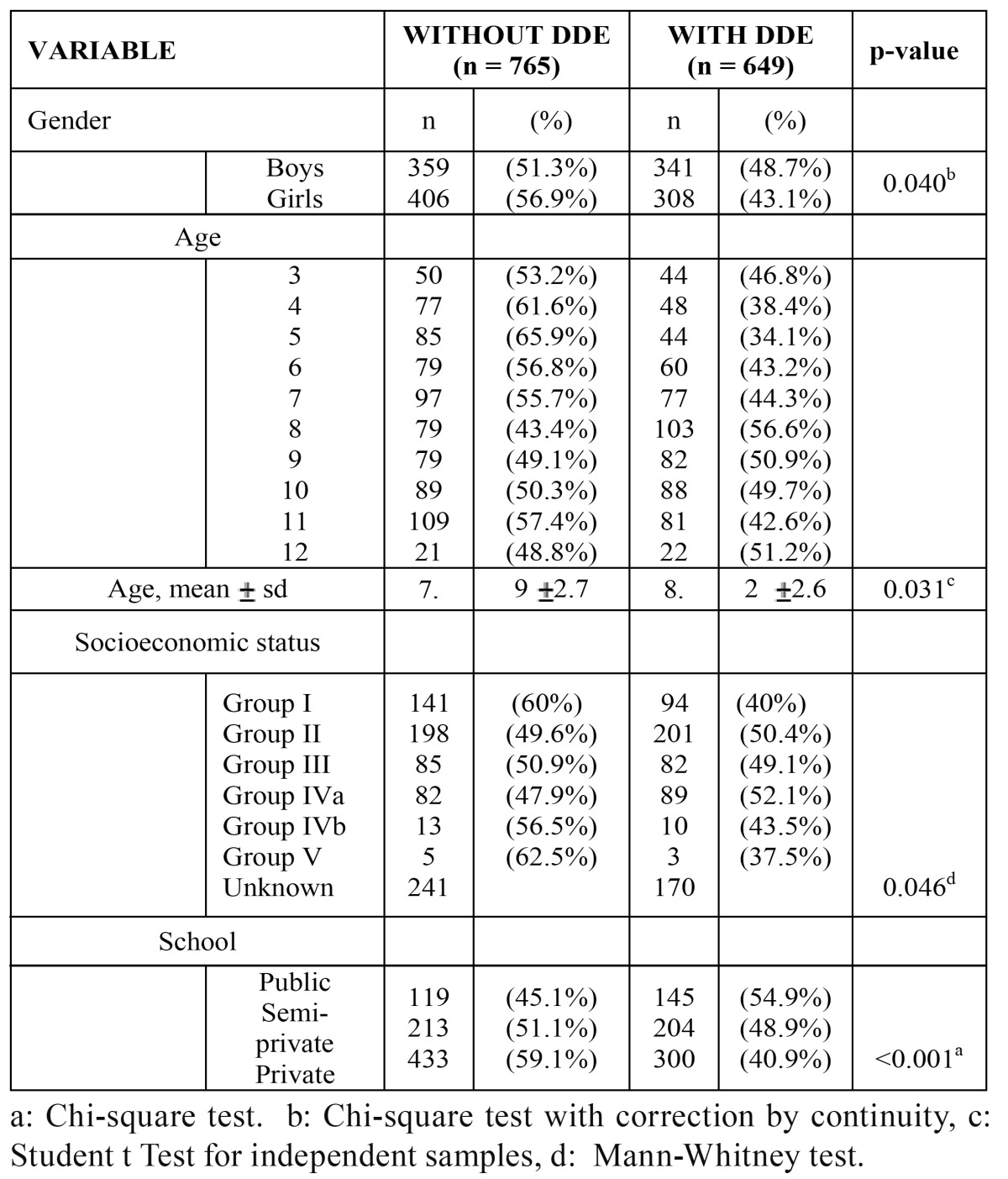
Number and percentage distribution of children with and without enamel defects (dde).

 Table 3 shows the prevalence of EH and EO in the primary and permanent dentition. Of the 31,820 primary and permanent teeth examined in the study, 699 (4.1%) primary and 1,232 (8.3%) permanent teeth had some form of DDE. EH was the least common type of DDE in both dentitions, whereas DIO was the most prevalent defect in primary teeth and DEO in permanent teeth (statis-tically significant differences; p<0.001).

**Table 3:**
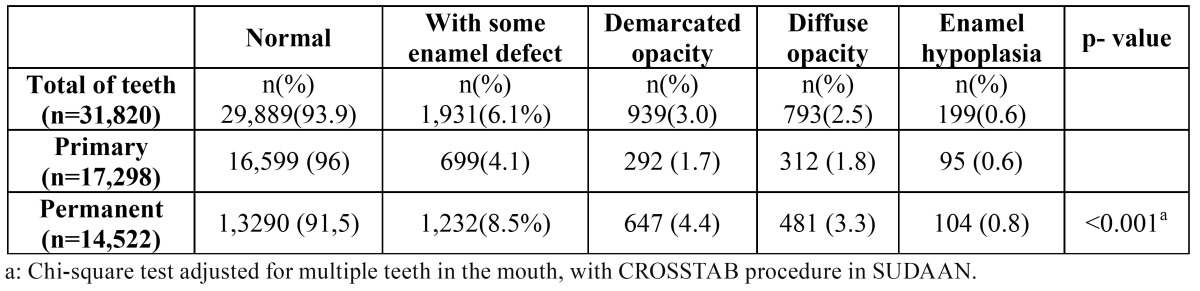
Prevalence of enamel hypoplasia and enamel opacity in primary and permanent teeth.

**Figure 2:**
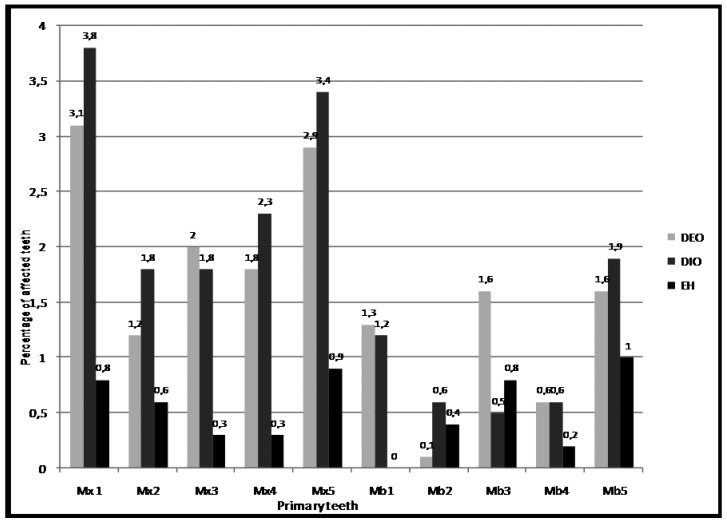
Prevalence of enamel hypoplasia (EH), diffuse opacity (DIO) and demarcated opacity (DEO), by primary tooth type. The differences in prevalence among the different tooth types are statistically significant “(p<0.001)” a Chi-square test adjusted for multiple teeth in the mouth, with CROSSTAB procedure in SUDAAN.

**Figure 3:**
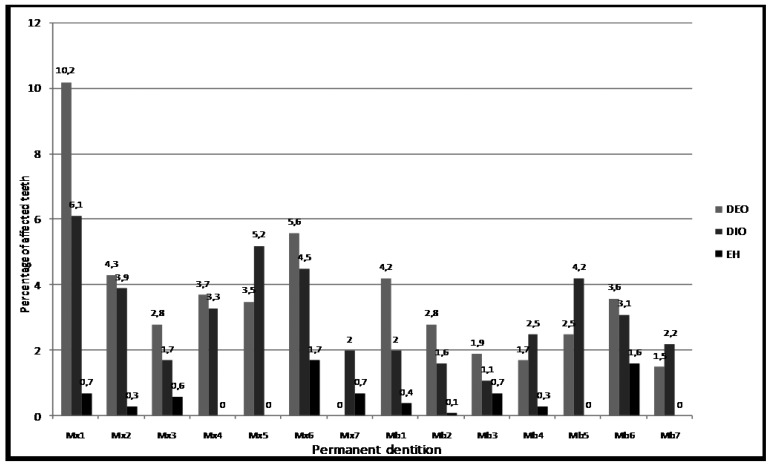
Prevalence of enamel hypoplasia (EH), diffuse opacity (DIO) and demarcated opacity (DEO) in the permanent dentition by tooth type. The differences in prevalence among the different tooth types are statistically significant “(p<0.001)” a. Chi-square test adjusted for multiple teeth in the mouth, with CROSSTAB procedure in SUDAAN.

Figures 2,3 illustrate the distribution of prevalence of EH, DIO and DEO by primary and permanent tooth types. Defects were most prevalent in: maxillary central incisors (Mx 1) and maxillary second molars (Mx 5) in primary dentition; and maxillary central incisors (Mx 1) and maxillary first molars (Mx 6) in permanent dentition. The differences in prevalence among the various types of primary and permanent teeth were statistically significant (p<0.001).

## Discussion

The results of the present study show that the prevalence rates of EO and EH in healthy children from a non-fluoride community in Spain lie within ranges previously reported for children in other developed countries (2,8,9,11-21). Permanent dentition was seen to be most affected by DDE, a finding in line with many research studies reporting lower prevalence in primary teeth (2,8,9,17). The increased DDE risk in permanent teeth is probably related to the critical period of amelogenesis between 0 and 2 years of age, when the child is particularly vulnerable to a number of common systemic conditions that can affect enamel devel-opment (2).

In addition, the fact that permanent maxillary central incisors, permanent first molars, are most commonly affected by DDE is consistent with most recent reports (2,7,17,19-21). The primary tooth most affected by DDE was the Mx 1, followed by Mx 5 and Mx 3. Previous authors have likewise found the incisor to be most affected (8,15,17), though in other studies, second primary molars were the most affected primary teeth (2,9,14).

Only a few epidemiological studies have examined the prevalence of DDE in primary teeth in healthy children from developed countries, indicating a range of prevalence from 24% to 49%. Our finding of 40.2% prevalence of DDE in primary teeth would fall within the mid-high range of reports in primary teeth (2,8,13-17), similar to the figure found in an Arab population (45.4%) (15). Some authors suggest that race could play a role in the appearance of DDE; a study of Asian children shows low prevalence (8), while the Australian Aboriginal population may have a particular susceptibility to DDE (28).

With regard to permanent dentition, our results showed figures of 52% of DDE in children examined, numbers that also fall within the medium-high range of published results (2,11,12,16,17,19-21), and resemble those obtained by Clarkson & O´Mullane (11) in Ireland (52.4%), or more recently, by Seow et al. (2) in children from a non-fluoride urban community of Australia (58%).

Moreover, our findings come to support the reported association between socioeconomic level and a high prevalence of DDE (7,12,29): enamel anomalies were more frequently found in children of lower-middle socioeconomic status (groups IV-V) enrolled in the public schools of our study.

Regarding gender, the greater prevalence of DDE in boys than in girls was similarly reported by Li et al. (8) in an Asian population and Farsi (15) in an Arab population.

The most common DDE in primary dentition was DIO, followed by DEO and EH. The association between fluoride and DIO has been demonstrated, hence the high prevalence of these defects in permanent teeth within communities with fluoridated drinking water (11,21). Primary dentition is thought to be less affected by fluorosis, as most primary teeth develop prenatally, when the fetus is partly protected from excessive fluoride levels. The community involved in our study has no natural or artificial fluoridation of drinking water. Chaves also obtained a greater prevalence of ODI in the primary teeth of children residing in a community with low fluoride levels (29), attributing the high percentage of ODI to maternal infections during pregnancy. The higher prevalence of primary dentition ODI obtained in our study, with respect to other research, might possibly be related to the fact that children were explored after brushing, with artificial light and teeth dried when in doubt with a gauze, which facilitated the diagnosis of such defects.

Most recent epidemiological investigations in primary dentition point to EH as the least common DDE (9,14,15), as we found. Therefore, primary teeth were affected more by qualitative lesions than by quantitative ones. This may be due to changes during maturation and calcification, rather than to phases of differentiation and secretion of the enamel matrix (30).

DEO was the most common dysplasia in permanent dentition, followed by DIO and EH. Most research in permanent teeth gives a higher frequency of DEO (2,7,11,20). Demarcated defects occur after damage to ameloblasts during the initial or final maturation phase, but the cells are able to recover and resume their normal function, suggesting that a transient origin such as infection may be involved. (2,12,18).

In conclusion, the high prevalence of developmental enamel defects found in primary teeth (40.2%) and permanent dentition (52%) among children in Spain suggests a need for further studies of the etiologic factors involved in this condition, in view of its clearly increasing prevalence and the fact that it entails limitations to life quality, technical difficulties for treatment, and the failure of current preventive measures.
